# Supplementary figures and images for: Oscillations in an artificial neural network convert competing inputs into a temporal code
Source: PLoS Comput Biol. 2024 Sep 11;20(9):e1012429. doi: 10.1371/journal.pcbi.1012429 (PMC11419396; doi:10.1371/journal.pcbi.1012429)

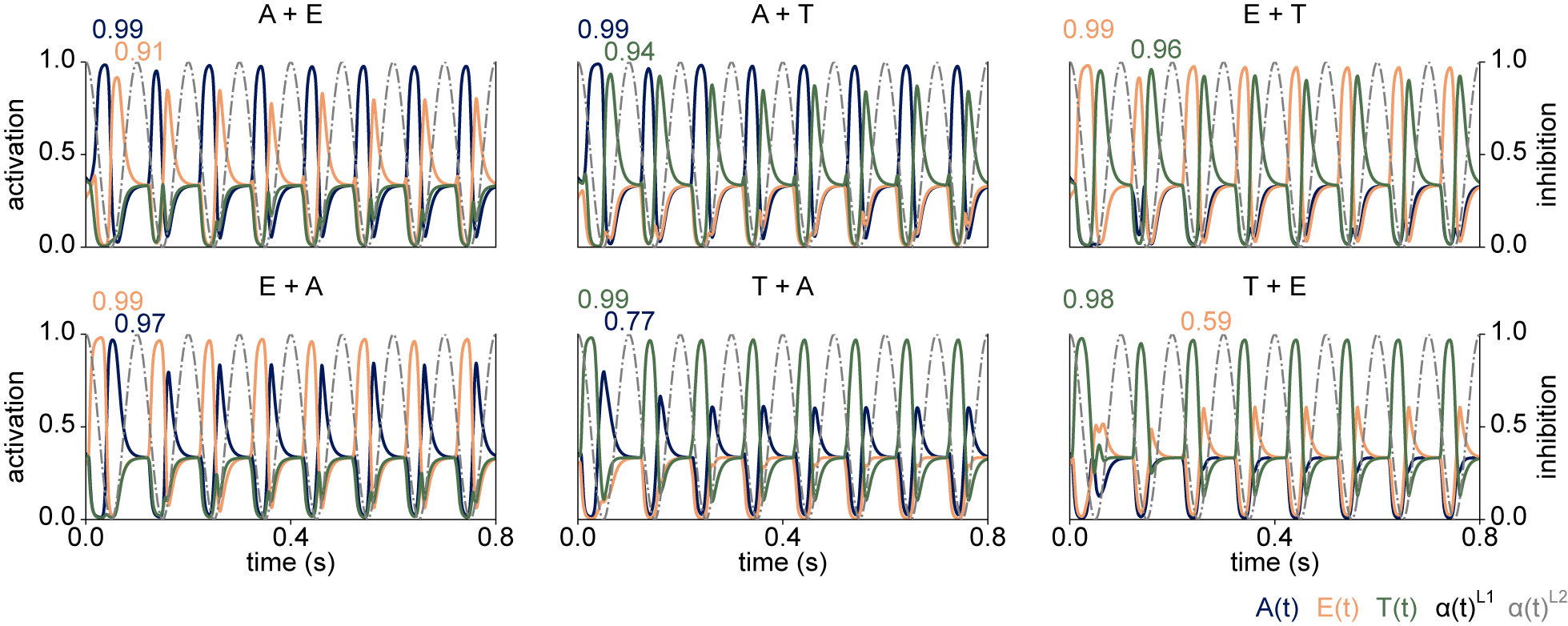

Supplement: S1 Fig — The first letter in the title is the one for which input gain has been increased. The coloured text indicates the read-out accuracy for each of the presented letters. (TIF) [file pcbi.1012429.s001.tif]

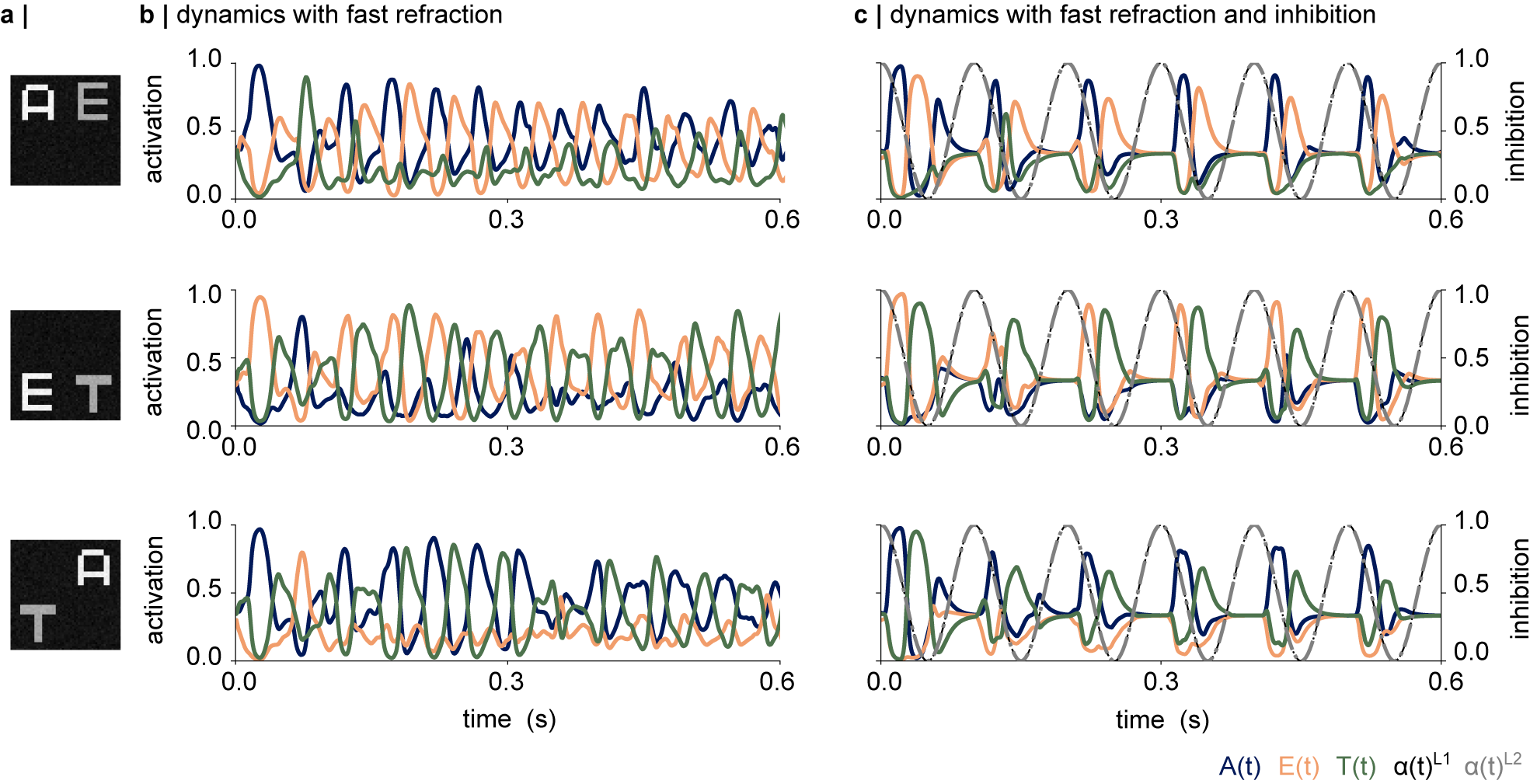

Supplement: S2 Fig — a | The experiment was performed on the same input combinations shown in Fig 6. b | Speeding up the refraction results in faster activations of the nodes corresponding to the two letters presented in the image. The dynamics again show some instability with a fluctuating amplitude and an occasional spurious activation of the letter that is not presented in the image. c | In presence of 10Hz inhibition, the attended letter is read out twice within one inhibitory cycle: once before, and once after the the unattended letter. Following that, the output again shows a temporal code with two items per cycle. (TIF) [file pcbi.1012429.s002.tif]

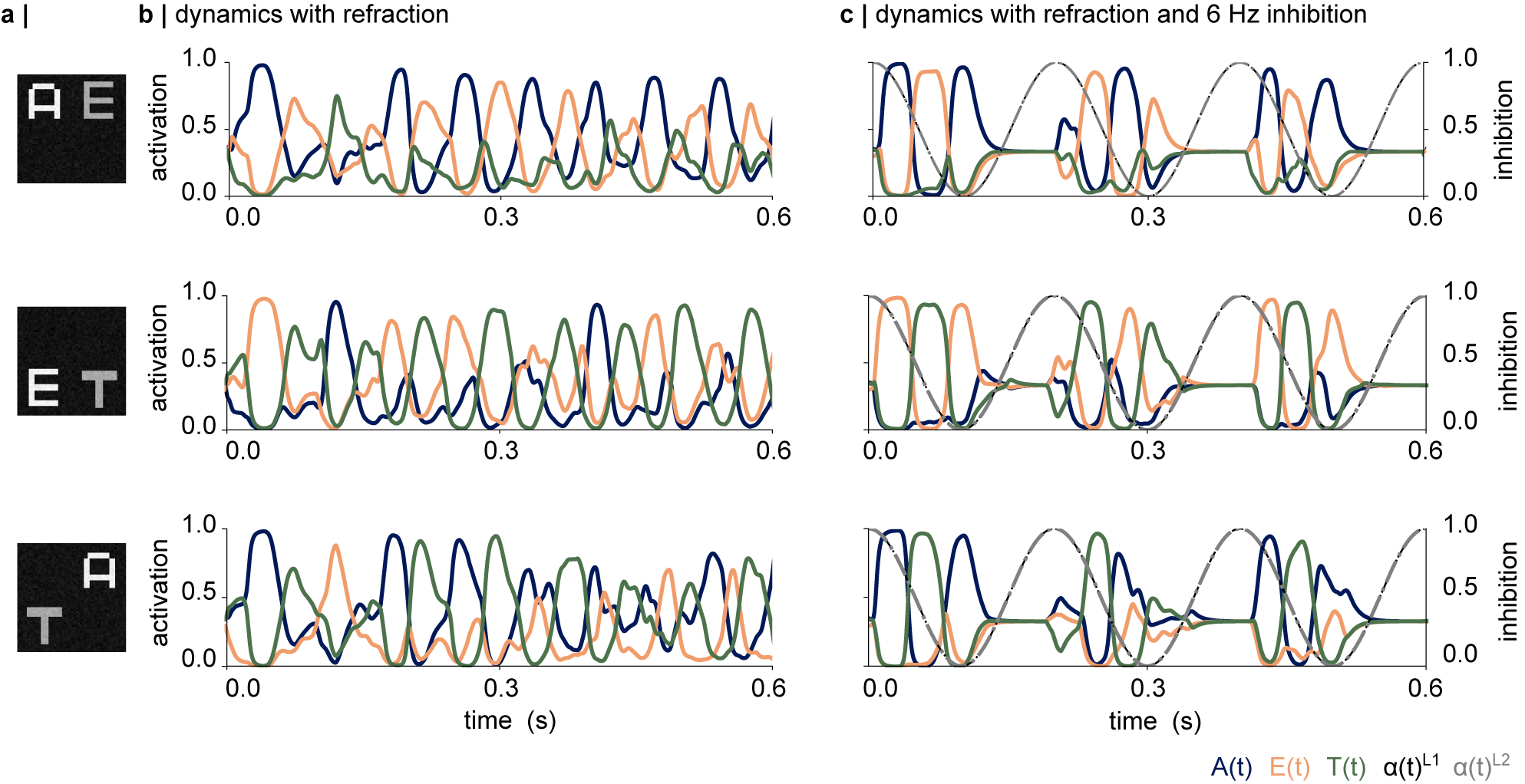

Supplement: S3 Fig — a | The experiment was conducted on the same input combinations shown in Fig 6 and S2 Fig. b | Dynamics with τr = 0.1s (same as Fig 6b). c | Slowing down the inhibition to 5Hz results in a temporal code with three items. Interestingly, in the second inhibitory cycle, the unattended letter seems to be read out first along the phase of the inhibition. This appears to be due to the brief, low-amplitude activation of the node corresponding to the attended item during the peak of the inhibition. The code stabilises again in the third inhibitory cycle. (TIF) [file pcbi.1012429.s003.tif]

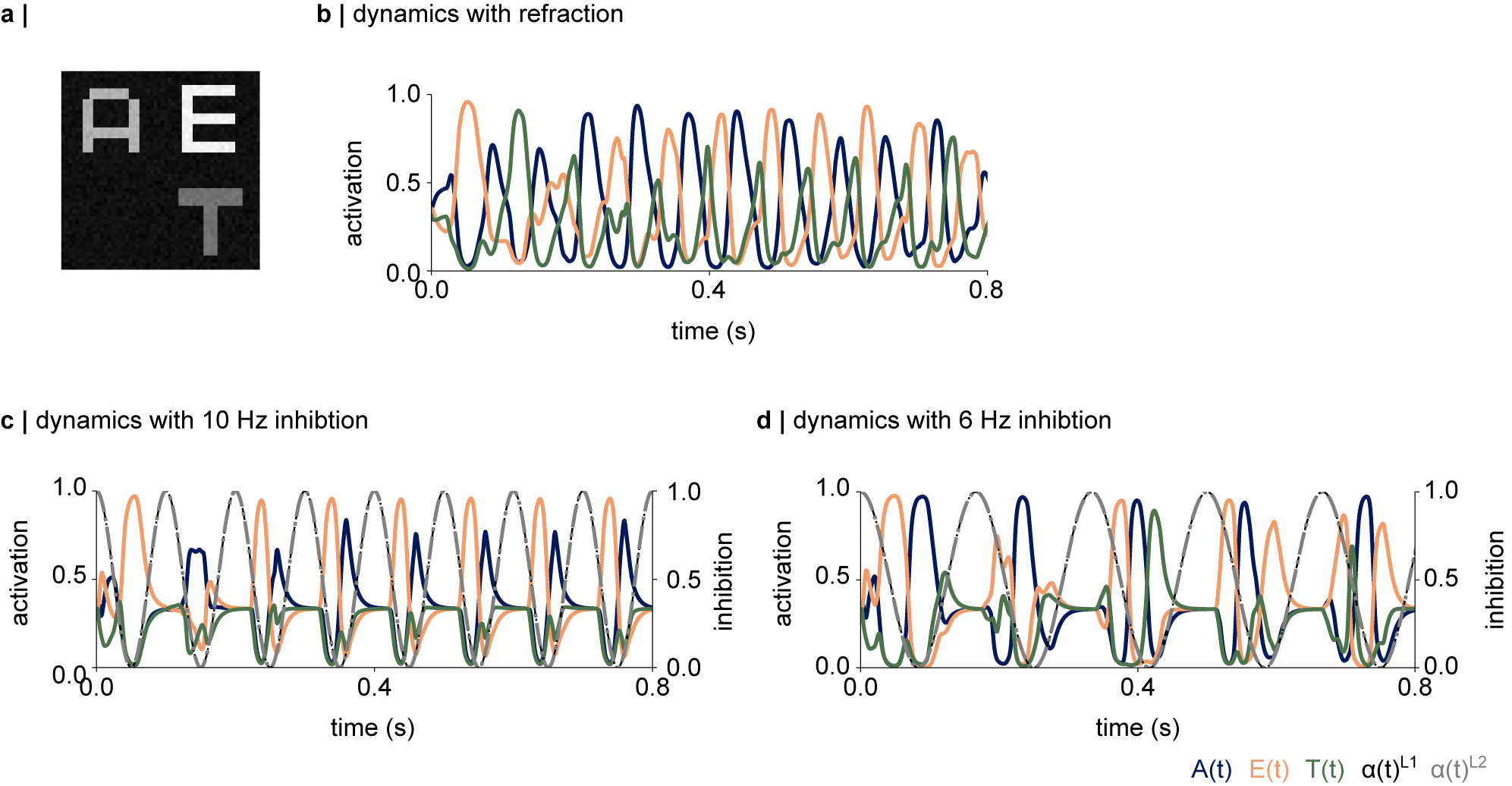

Supplement: S4 Fig — a | The image with three inputs. “E” had the highest luminance, followed by “A”, and “T”. b | In absence of inhibition, the three output nodes corresponding to the inputs activate in succession. The amplitude of the unattended “T” is notably reduced compared to “E” and “A”. c | With a 10Hz inhibition, as used in the previous simulations, “E” is read out as the only letter in the first cycle, “A” activates in the second cycle. Following that, a stable temporal code with “E” and “A” is produced in each cycle. d | With a slower inhibition of 6Hz, the three letters activate in the first and second cycle, ordered based on their luminance. However, in the second and forth cycle, the temporal code only consists of the letters “E” and “A”, whereby “E” activates twice. In the fifth cycle shown here, “T” activates briefly after “E” is activated. (TIF) [file pcbi.1012429.s004.tif]

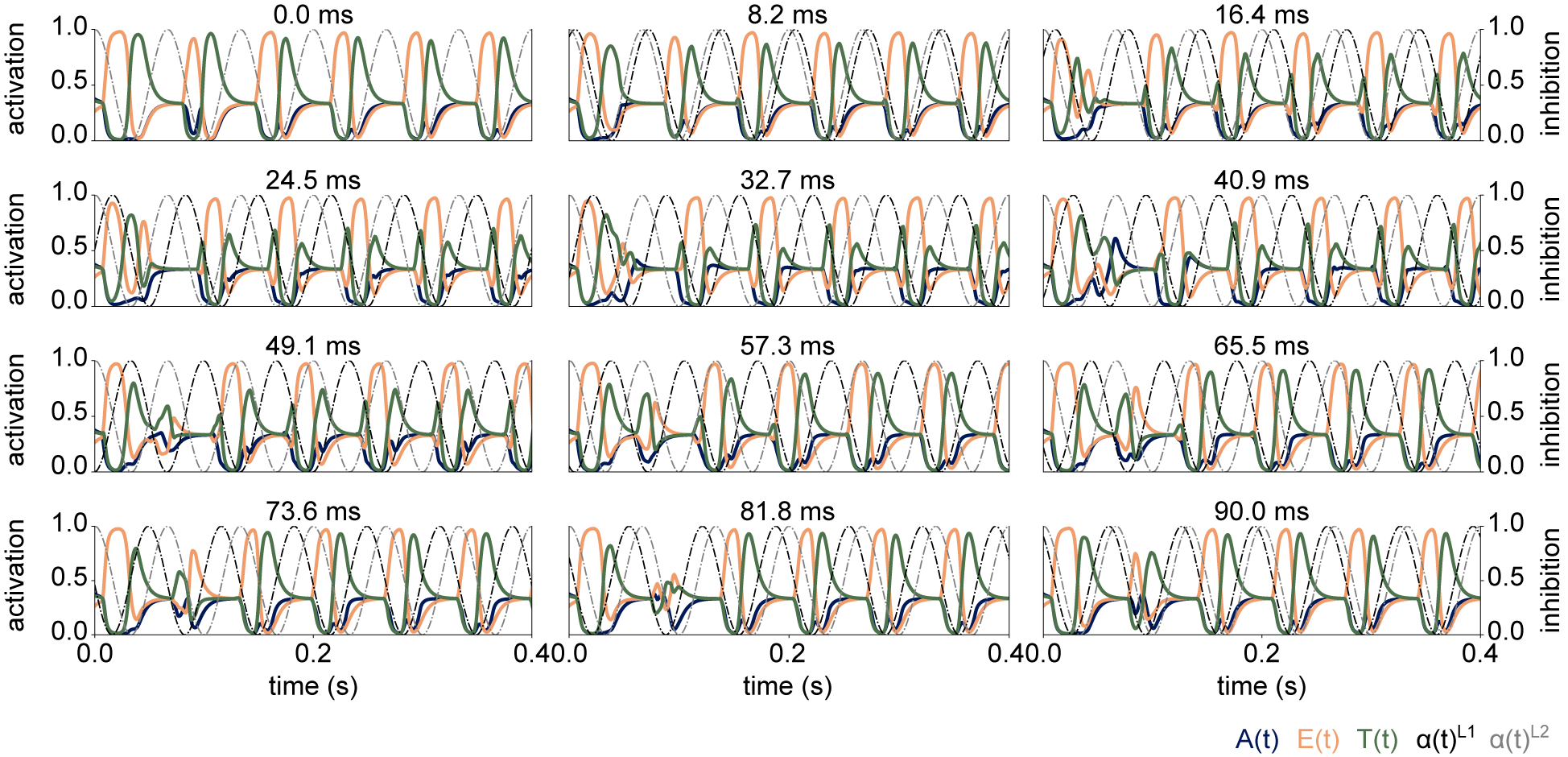

Supplement: S5 Fig — The temporal code is more strongly affected when shifting the phase of the inhibitory oscillation layer 1 compared to layer 2 (compare Fig 8). Notably, shifting the phase of the inhibition in the first layer mainly seems to affect the activations in the first two cycle of the inhibition (see 16.4–81.8ms), however, the temporal code often appears to recover within three cycles. (TIF) [file pcbi.1012429.s005.tif]
