# Supplementary material for: Oscillations in an artificial neural network convert competing inputs into a temporal code
Source: PLoS Comput Biol. 2024 Sep 11;20(9):e1012429. doi: 10.1371/journal.pcbi.1012429 (PMC11419396; doi:10.1371/journal.pcbi.1012429)
Supplement: Attachment — Submitted filename: review Plos CB PCOMPBIOL-D-24-00669.pdf [file pcbi.1012429.s006.pdf]

Oscillations in an Artificial Neural Network Convert Competing Inputs into a Temporal Code: Review

The authors present an ANN to which they add biologically-inspired dynamics to implement inhibition through alpha-oscillations. They train their dynamical ANN to decipher between pairs of simultaneous stimuli (pairs of letters) to mimic the need of object based attention when presented with several stimuli. That's a nice concept and it makes sense.
However, my main concern is regarding the stimuli themselves: from what is displayed in the figures, they use three letters, A, E and T, printed in white over a black background, but the problem is that each letter seems to always be situated in the same, non overlapping part of the total input image (i.e. A is in the top right corner, E in bottom left and T in the top left). So the question is whether the ANN learns to decipher between the letters or just the region of the image that has some white pixels in it..? Also I am a bit confused about the training procedure..

Question 1: Could you show what happens when you change the stimuli's position on the image, and/or what happens if you repeat twice the same stimuli in different positions on the same input image? Also what happens if you put one of the letters in the bottom left corner of the image? Because, to follow your example, maybe the apple and passionfruit are not always exactly at the same place on the supermarket shelves and yet, you recognize them regardless…
In case I misunderstood and the letters are not always in the same position and this is just for the example images you choose you need to clarify that..

Question 2: Could you introduce some noise on the inputs, i.e. not have exactly the same shape every time for the same letter but small variations? How does that affect your results? Without it it is hard to know if your results can generalize at all…

Question 3: Could you quantify the results accuracy that you show for all the different experiments? i.e. give the readout accuracy or how much overlap/no overlap for the stimuli distinction..

Question 4: In figure 6, could you add an example of A+T (I know you give examples of all combinations in fig. S1 but it would be nice to have it here). And also, what happens with the three simultaneous stimuli?

Question 5: Could you explain the training procedure in the methods section? Also you should describe clearly your training set (cf. question 1). There is just a very brief explanation in the legend of figure 2 and nothing in the main text.. Also what does it mean that the MSE is "approaching 0"? How much is it? Is it on the training or on the test set? Is there a test set?

Minor comments:

- Typo: you always refer to figure 2 as figure 3…
- In figure 2, I would change the labels order to follow the order of the text: 2d->2b, 2b->2c and 2c->2d.
- Figure 3 and results section 3.1 (bottom of page 6): can you make more explicit the parts without alpha inhibition vs when there is? i.e. in the text specify explicitly that

you first train without and in the figure put some titles or something to differentiate the sides without and with alpha (similar as what you do in figure 4).

- Figure 7: I’m not sure what it is but it’s not very easy to read… Maybe you need to put an inset on time or something like that so it makes it easier to see the sequential activations of the neurons…
- Figure 8: this one is also a bit hard to read… Maybe make everything larger? Or I’m not sure what you should do exactly but try to make it more digest if you can…
- Also generally for all figures: everything is very small (titles, legends etc.), maybe you can increase a little bit the fonts you use to make it easier for the reader..
- There’s a typo in the intro page 2, third paragraph “object recognition has been argued ***to*** have a limited capacity.
