# Supplementary material for: Oscillations in an artificial neural network convert competing inputs into a temporal code
Source: PLoS Comput Biol. 2024 Sep 11;20(9):e1012429. doi: 10.1371/journal.pcbi.1012429 (PMC11419396; doi:10.1371/journal.pcbi.1012429)
Supplement: Attachment — Submitted filename: Deucker_etal_PLOSReview.pdf [file pcbi.1012429.s007.pdf]

Review of the paper “Oscillations in an artificial neural network convert competing inputs into a temporal code” by Duecker et al.

The efficient processing of multiple, competing inputs is a fundamental task in computational neuroscience and machine learning. In this work, Duecker et al. implement a brain-inspired mechanism to address such task in the context of image classification in an artificial neural network. Such mechanism consists in enforcing, after training, a dynamics of nodes activations that mimick alpha-oscillations and rythmic inhibition in the brain. When an image containing multiple stimuli is presented, such dynamics allow for an alternating representation of the competing inputs, effectively embedding a serial, multiplexed neural code. I find such mechanism a simple but elegant idea, well motivated by biological findings, and the results exposition is convincing in supporting its effectiveness. I do have some comments and suggestions that might help in improving the clarity of the exposition and the reproducibility of the results, but overall my impression is highly positive. I find that this paper is a great contribution to the growing literature of models at the interface between computational neuroscience and machine learning and of potential interest for both communities.

Main suggestions:
I found the explanation of the architecture of the deep neural networks employed a bit obscure:

- First, I guess there is a misprint in the Methods section introduction, where it is stated that a 2-layer architecture was employed, but then a three-layer one was discussed below and illustrated in the figure.
- Second, it is not clear to me the sentence “A weight matrix of size 28 × 28 was applied to the input (56 × 56) with a stride of 28, such that each node in the first layer received 4 × 28 × 28 inputs, ensuring representational invariance across the quadrants in the input.”. If I understood well from the code, the first layer is supposed to be a conv2d layer with 64 output features, effectively learning a single 28x28 kernel for each feature, followed by a sum operation. I suggest unpacking and clarifying this passage.
- Below Eq. (1) is written that each input is a linear sum of activations in the previous layer, $\sum w_{ij} z_j$, but shouldn’t be h instead of z?
- It is not well explained how the post-training dynamics is propagated across layers, especially because different types of propagations (e.g., with phase delay) are used later in the manuscript.

Relatedly, it would be interesting to check the robustness and generalizability of the results across a range of similar architectures. In particular, in the architecture described in the manuscript, few possible images are allowed (3 letters x 4 quadrants), and the kernel is perfectly adapted to the size of the image (28x28), and a large stride is employed, effectively conveying independent input for each quadrant. This could potentially result in overfitting. I think it would be instructive to explore architectures with smaller, more common kernel sizes and stride (e.g., 3x3 or 5x5) to see how results generalize when the first layer learns more elementary features. In particular, I expect the second layer dynamics in Fig. 7 to exhibit less segregation, but this is just a guess.

Minor comments:

- If the task is classification, why using the mean squared error as training objective (fig. 2c)?
- There seems to be some inconsistency in Figure referencing, e.g., in page 3 both citations should be Figure 2, and similary in page 5, and 6 (last reference before the last paragraph). In page 8, paragraph 3.3.1, the reference should to Fig 5b.
- In Eq. (8), h_j seems to be a scalar, there is no need to define the Hadamard product
- The words refractory and relaxation dynamics are used interchangeably, I would stick to one choice to avoid confusions
- I really enjoyed reading the discussion about the possible future directions on the neuroscientific and machine learning side. In this case, the dynamics was imposed after training; I wonder whether the authors could instead comment on possible training mechanisms that would give rise to such dynamics?
