# Supplementary material for: Oscillations in an artificial neural network convert competing inputs into a temporal code
Source: PLoS Comput Biol. 2024 Sep 11;20(9):e1012429. doi: 10.1371/journal.pcbi.1012429 (PMC11419396; doi:10.1371/journal.pcbi.1012429)
Supplement: Attachment — Submitted filename: kduecker_response_reviewers.pdf [file pcbi.1012429.s008.pdf]

Revisions

For the revisions, the reviewer's original comments are marked in italic, our response to the reviewer is marked in bold, and the added text passages are written in normal font. In the revised manuscript, we have marked the edits based on the comments by reviewer 1 in blue, reviewer 2 in green, and reviewer 3 in pink.

Reviewer 1

We thank the reviewer for their time revising our manuscript and their positive feedback. All edits related to the reviewer's comments are indicated in blue in the revised manuscript.

There is a broad range of temporal dynamics in the neural system, and these temporal dynamics are thought to have an important function in the representation of information, especially multiple competing information. In this manuscript, the authors trained the ANN to recognize letters, and with adding of temporal dynamics in the hidden layer, the ANN was able to read out sequentially for two letters presented at the same time. Moreover, the sequence of readout is along the phase of inhibitory oscillations. The results of the study proved that the inhibitory oscillations help to segregate the information in time. Overall, this study is a timely and insight-providing study that demonstrates the positive effects of oscillations on information readout. It was a pleasure to read such a well-designed and well-written study, and I think that both the neuroscience and machine learning fields will have interest in the results of this study, and I would like to see it published.

I have 3 small suggestions that the author might consider.

1. I would like the authors to discuss how the findings of this study contribute to our understanding of how the neural system works.

The main contribution of our algorithm is that we test whether inhibitory oscillations serve to support visual processing. Neuronal alpha oscillations have long been known to be inhibitory, but their relevance for neuronal computation is debated. Specifically, we test whether inhibitory oscillations allow a neural network to overcome computational bottlenecks when processing multiple inputs.

Moreover, our algorithm is rooted in insights from neuroscientific and cognitive studies of the visual system, which have revealed that object recognition underlies both parallel and serial processes.

Lastly, we outline predictions based on our simulations that can be tested using electrophysiological approaches.

Please see below for details.

l. 343:

A key contribution of our approach is that we translate conceptual ideas based on neuroscientific studies into a computational model.

Our network embraces two key properties of visual perception: parallelisation and segregation. Prior work has shown that simple visual features can largely be processed in parallel [5, 30, 29], while object recognition has been demonstrated to be supported by serial processes [15, 24]. This indicates a bottleneck problem which has been argued to arise from the converging hierarchical structure of the visual system [3, 11, 24, 29]. A similar bottleneck problem arises in our non-dynamic network (Figure 5).

Also see ll. 357

Our algorithm further draws from evidence for phase-coding observed in recordings from the rodent and human hippocampus, whereby spiking activity has been shown to be modulated along the phase of ongoing theta oscillations (4-8 Hz [13, 16, 23, 26, 27]). The order in which a sequence of inputs has been experienced, has further been proposed to be preserved in the spiking activity [13, 23] but see [22]. We here demonstrate how the visual system may utilise a similar mechanism based on inhibitory alpha oscillations to support object recognition.

As outlined in 2 Methods, we tuned the hyperparameters in our simulations to resemble oscillatory dynamics observed in electrophysiological recordings. The rise time of the activation τ_h was chosen based on the membrane time constant of excitatory neurons (10 - 30 ms) [4]. The activation period of an individual letter within a temporal code was 23-30 ms (Figure 6b and c), i.e. 35 to 40 Hz. This corresponds to the period of gamma oscillations, which have been proposed to be involved in the feedforward processing of visual information [1, 2, 17]. As such, our algorithm is strongly linked to the idea that visual processing is modulated by an interplay of gamma and alpha oscillations [17, 12].

The involvement of these oscillations in organising visual processing is backed by a rich body of literature.

And ll. 389

In addition to the explored computational benefit of inhibitory oscillations in visual processing, a key contribution of our study is that we demonstrate two testable predictions.

2. I would like to know if adding other frequencies of oscillations to the hidden layer by changing the time parameter would produce similar results to this study? In other words, could the authors discuss the similarities and differences between brain information processing and ANN information processing, especially in the time scales.

This is a great point that has prompted us to do further simulations presented in Figure S2 and S3. We further explore the role of the frequency of the inhibition and refraction in the discussion session.

ll. 261

We hypothesised that speeding up the refraction would allow an increase of the number of items within the temporal code. This was tested by repeating the simulations shown in Figure 6 with $\tau_r = 0.05$. However, while a reduced time constant for the refraction did result in a faster activation of the two nodes corresponding to the letters in the image (Figure S2b), only the first inhibitory cycle showed three activations, whereby the attended letter is read out before and after the unattended one (Figure S2c). A faster refraction was further associated with an overall reduced amplitude, and an occasional activation of the output node corresponding to the letter that was not presented in the image. Overall, decreasing the time course of the refraction did not seem to offer a stable solution for increasing the number of items in the temporal code.

Reducing the frequency of the inhibitory oscillation to 5 Hz, lead to a robust temporal code with three activations per cycle of the inhibitory oscillation (Figure S3c). These simulations do however still show a activation to the letter not presented (Figure S3c top and middle panel). For the presented algorithm, slowing down the inhibition appears to be more effective to include more items into the phase code than speeding up the refraction.

And ll. 363

As outlined in 2 Methods, we tuned the hyperparameters in our simulations to resemble oscillatory dynamics observed in electrophysiological recordings. The rise time of the activation τ_h was chosen based on the membrane time constant of excitatory neurons (10 - 30 ms) [4]. The activation period of an individual letter within a temporal code was 23-30 ms (Figure 6b and c), i.e. 35 to 40 Hz. This corresponds to the period of gamma oscillations, which have been proposed to be involved in the feedforward processing of visual

information [1, 2, 17]. As such, our algorithm is strongly linked to the idea that visual processing is modulated by an interplay of gamma and alpha oscillations [17, 12].

3. There is a small typo, the second line of 2.1 on page 3. Should it be Figure 2a?

Thank you, we have corrected that.

Reviewer 2

We thank the reviewer for their time and thorough evaluation of our manuscript. All revisions in the manuscript based on the reviewer's suggestion are marked in green.

The authors present an ANN to which they add biologically-inspired dynamics to implement inhibition through alpha-oscillations. They train their dynamical ANN to decipher between pairs of simultaneous stimuli (pairs of letters) to mimic the need of object based attention when presented with several stimuli. That's a nice concept and it makes sense. However, my main concern is regarding the stimuli themselves: from what is displayed in the figures, they use three letters, A, E and T, printed in white over a black background, but the problem is that each letter seems to always be situated in the same, non overlapping part of the total input image (i.e. A is in the top right corner, E in bottom left and T in the top left). So the question is whether the ANN learns to decipher between the letters or just the region of the image that has some white pixels in it..? Also I am a bit confused about the training procedure..

Question 1: Could you show what happens when you change the stimuli's position on the image, and/or what happens if you repeat twice the same stimuli in different positions on the same input image? Also what happens if you put one of the letters in the bottom left corner of the image? Because, to follow your example, maybe the apple and passionfruit are not always exactly at the same place on the supermarket shelves and yet, you recognize them regardless... In case I misunderstood and the letters are not always in the same position and this is just for the example images you choose you need to clarify that..

We should have indeed explained this better in the previous version of the manuscript. Each letter was indeed presented in one each the four quadrants. We have updated Figure 2b to reflect this. Moreover, we have used different input images with the letters at different locations for the simulations in Figure 5 and Figure 6 to clarify this. Also see l. 114

In each epoch, the network was trained on 132 images, whereby each letter appeared at each location on the image.

Question 2: Could you introduce some noise on the inputs, i.e. not have exactly the same shape every time for the same letter but small variations? How does that affect your results? Without it it is hard to know if your results can generalize at all...

Following the reviewer's suggestion, we have now added some (low amplitude) noise to the images for training and testing, as depicted in Figure 2b, Figure 5a and Figure 6a. As such, all simulations performed with dynamical ANN were performed using stimuli the network had not seen before.

Question 3: Could you quantify the results accuracy that you show for all the different experiments? i.e. give the readout accuracy or how much overlap/no overlap for the stimuli distinction..

We have added the read-out accuracy to Figure S1. Please also see l. 254

The maximum read out accuracy (activation) for each letter is indicated in each plot. As the activations were calculated using a softmax function, a value of 0.99 (e.g. for letter "A" in top left panel), indicates that the network is 99% certain about the presence of the letter "A" while the remaining 1% are shared between letters "E" and "T". While the response to "E" in the combined "T" and "E" input is notably reduced compared to the other experiments (Figure S1 bottom right), the network still achieves a read out accuracy of 0.59, well above the chance value of 0.33. The simulations show that the network is able to segregate all input combinations in the test set.

Question 4: In figure 6, could you add an example of A+T (I know you give examples of all combinations in fig. S1 but it would be nice to have it here). And also, what happens with the three simultaneous stimuli?

We have added a plot showing a combined input of A and T to Figure 6. We also explore a simulation with 3 simultaneous inputs in Figure S4, as described in the manuscript ll. 273

Following these tests, we investigated whether the network could generate a temporal code representing all three stimuli. Figure S4a shows the exemplary input, which was generated by multiplying the attended letter ("E") by 1.2, the unattended letter("T") by 0.8, and "A" with 1. After adding the noise, the image was scaled to the luminance range from 0 to 1. We used the original settings for the dynamics with $c = 10$, $s = 0.1$, $\tau_h = 0.01$, and $\tau_r = 0.1$. Indeed, the refraction without inhibition allow the network to dynamically activate each letter in the input, albeit with varying amplitude and activation period (Figure S4b). Generating a temporal code with all three items, however, proved to be challenging. Introducing a 10 Hz inhibition resulted in "E" and "A" being read out in the first and second cycle of the inhibition, respectively, after which the network produced a code with two items ("E" and "A") in each cycle (Figure S4c). Slowing down the inhibition to 6 Hz resulted in a temporal code with three items in two out of the five cycles shown here,

however, the network often activated the output node corresponding to "E" after reading out "A" (Figure S4d). In sum, while the network was able to produce a stable temporal code with two inputs, it was not trivial to produce a code with three stimuli. We will explore the biological relevance of increasing the number of items in the temporal code in the Discussion.

In the discussion section, we outline that we were predominantly interested in generating a temporal code with two items, due to a hypothesised link between the multiplexing and saccadic reviewing. This paragraph is marked in blue as it mainly addresses a comment by reviewer 1.

ll. 380

In our model, the number of items in the temporal code could be increased by reducing the frequency of the inhibitory oscillation (Figure S3). This simulation suggests that the visual system may slow down alpha oscillations in anticipation of complex visual inputs. So far, however, only an increase in alpha frequency has been linked to visual detection and processing speed in temporal attention paradigms [7, 25]. Moreover, multiplexing by alpha oscillations has been proposed to support saccadic previewing [11]. According to this model, the first item in the temporal code represents the stimulus that is currently fixated, while the second item may represent the goal of the next saccade [11]. Therefore, while changing the dynamics may be relevant for computational goals in the dynamical ANN, we believe that the temporal code with two items organised by inhibitory 10 Hz oscillations may capture the dynamics of visual cortex and associated conceptual models more accurately.

Question 5: Could you explain the training procedure in the methods section? Also you should describe clearly your training set (cf. question 1). There is just a very brief explanation in the legend of figure 2 and nothing in the main text.. Also what does it mean that the MSE is "approaching 0"? How much is it? Is it on the training or on the test set? Is there a test set?

The reviewer is correct that we used the same stimuli for training and testing in the previous version of the manuscript. Now that we are using images with noise, we generated a new set of noisy stimuli that were not part of the training set. We have updated the section on network training in ll. 109

The training set consisted of three letters, presented in one of four quadrants in the image. After adding gaussian-distributed noise ranging from 0.01 to 0.25, each input was normalised by its maximum value, such that the luminance in each image ranged between 0 and 1 Figure 2b). The weights of the network were

initialised according to a uniform distribution within the range $[-x, x]$, where $x = \sqrt{\frac{6}{n_{\text{in}}+n_{\text{out}}}}$ with n_{in} and n_{out} being the number of inputs and outputs to the current layer, respectively (Glorot initialization) [9]. The Adam optimiser was chosen to minimise the cross-entropy loss using stochastic gradient descent [18].

In each epoch, the network was trained on 132 images, whereby each letter appeared at each location on the image. The network weights were learned by backpropagating the error through the network layers (as mentioned above, the bias term was fixed at $b = -2.5$). All experiments reported in 3 Results were conducted on a test set of noisy images with letters "A", "E", and "T" the network had not seen during training.

Minor comments:

- Typo: you always refer to figure 2 as figure 3...

Thank you, we have corrected this.

- In figure 2, I would change the labels order to follow the order of the text: 2d->2b, 2b->2c and 2c->2d.

The labels in Figure 2 are now in accordance with the order in which they appear in the text.

- Figure 3 and results section 3.1 (bottom of page 6): can you make more explicit the parts without alpha inhibition vs when there is? i.e. in the text specify explicitly that you first train without and in the figure put some titles or something to differentiate the sides without and with alpha (similar as what you do in figure 4).

We have added a title to Figure 3a and f to make explicit which column shows the frequency and amplitude with and without inhibition.

- Figure 7: I'm not sure what it is but it's not very easy to read... Maybe you need to put an inset on time or something like that so it makes it easier to see the sequential activations of the neurons...

We have updated Figure 7 and the associated text and caption to describe the parallel representation and segmentation along the layers of the neural network. We further chose to only plot 400 instead of 600 ms, to increase the size of the line plots in the top panel.

See l. 288

The top panels in Figure 7a and b indicate how strongly the hidden representations to the combined input correspond to the neural representations of both letters individually.

And ll. 292

A similarity value of $s_E(t) = 1$ in Equation (8) indicates that all hidden nodes corresponding to the individual letter (E and T), are activated by the current image showing both letters simultaneously.

In the first layer, the normalised dot product indicates that the nodes representing both "E" (orange trace) and "T" (green trace) activate in parallel, in anti-phase to the inhibitory oscillation (Figure 7a top panel). The network also appears to activate the hidden representation of "A", albeit to a lesser extent (blue trace). Indeed, the time course of the activations in each node (Figure 7a, bottom panel) demonstrates that almost all nodes in the first layer activate during the excitatory cycle of the oscillation. This indicates that the first layer represents the two presented letters in parallel.

In comparison, the activations in the second layer demonstrate that the nodes responding to each letter are activated in a sequence: the normalised dot product between the current representations and the activations to an individual letter “E” precede the ones corresponding to letter T (green trace, Figure 7b). The bottom panel in Figure 7b indicates that a smaller fraction of the network is activated at each time point, and the successive activation of the hidden nodes can be observed. Finally, Figure 7c shows the read-out in the output layer, confirming that the representations of "E" and "T" are fully separated during the excitatory cycle of the inhibition (also see Figure 6c).

In sum, our simulations show how integrating dynamics driven by excitation and refraction enables a fully connected neural network to multiplex simultaneous inputs – a task it has not been trained on explicitly. This mechanism is further stabilised by pulses of inhibition at 10 Hz, akin to alpha oscillations in the human visual system.

- Figure 8: this one is also a bit hard to read... Maybe make everything larger? Or I'm not sure what you should do exactly but try to make it more digest if you can...

We have changed the layout from 3 to 4 rows with 3 plots per row to increase the size of the plots.

- Also generally for all figures: everything is very small (titles, legends etc.), maybe you can increase a little bit the fonts you use to make it easier for the reader..

We have increased the font sizes in all plots as well as the width of the line graphs in each Figure.

*- There's a typo in the intro page 2, third paragraph "object recognition has been argued *to* have a limited capacity.*

This typo is now corrected.

Reviewer 3

We thank the reviewer for their time and effort reviewing our manuscript, and the positive feedback. All revisions related to the reviewer's comment are marked in pink in the revised version of the manuscript.

The efficient processing of multiple, competing inputs is a fundamental task in computational neuroscience and machine learning. In this work, Duecker et al. implement a brain-inspired mechanism to address such task in the context of image classification in an artificial neural network. Such mechanism consists in enforcing, after training, a dynamics of nodes activations that mimick alpha-oscillations and rythmic inhibition in the brain. When an image containing multiple stimuli is presented, such dynamics allow for an alternating representation of the competing inputs, effectively embedding a serial, multiplexed neural code. I find such mechanism a simple but elegant idea, well motivated by biological findings, and the results exposition is convincing in supporting its effectiveness. I do have some comments and suggestions that might help in improving the clarity of the exposition and the reproducibility of the results, but overall my impression is highly positive. I find that this paper is a great contribution to the growing literature of models at the interface between computational neuroscience and machine learning and of potential interest for both communities.

Main suggestions: I found the explanation of the architecture of the deep neural networks employed a bit obscure:

- First, I guess there is a misprint in the Methods section introduction, where it is stated that a 2-layer architecture was employed, but then a three-layer one was discussed below and illustrated in the figure.

- Second, it is not clear to me the sentence "A weight matrix of size 28 × 28 was applied to the input (56 × 56) with a stride of 28, such that each node in the first layer received 4 × 28 × 28 inputs, ensuring representational invariance across the quadrants in the input.". If I understood well from the code, the first layer is supposed to be a conv2d layer with 64 output features, effectively learning a single 28x28 kernel for each feature, followed by a sum opera3on. I suggest unpacking and clarifying this passage.

We have updated the paragraph in "2.1. Network architecture" to make clear that the network is indeed a network with only two hidden layers, whereby the convolutional kernel is only used to ensure weight-sharing, meaning competition, between the quadrants. This was important, as the conventional approach to implementing a fully connected network, i.e. flattening the image and connecting each

pixel in the input to the first hidden layer would have resulted in the emergence of four networks, one for each quadrant.

See ll. 90

The inputs to the network were images of size 56×56, each showing one of three letters ("A", "E", and "T"), presented in one of the image's quadrants (Figure 2b). We aimed to show that integrating oscillatory dynamics into the hidden layers would allow the network to overcome computational bottlenecks when processing an image presenting two letters at the same time. Therefore, we implemented competition between the quadrants by applying a weight matrix of size 28×28 to the input with a stride of 28. The results of the convolution between each quadrant and the weight matrix were then summed in each hidden node. To make the network dynamics tractable, we refrained from using a conventional CNN architecture, and instead used the convolutional kernel merely to implement weight sharing between the quadrants (but see 4 Discussion).

- Below Eq. (1) is written that each input is a linear sum of activations in the previous layer, ... but shouldn't be h instead of z?

Thank you, we have updated this, see l. 101

The input z_j arises from the activation in the previous layer according to $z_j = \sum_i w_{ji} h_i$, with w_{ji} being the weight matrix connecting nodes i and j, and h_i being either the pixels in the image (for hidden layer 1) or the activations in the first hidden layer (for hidden layer 2).

- It is not well explained how the post-training dynamics is propagated across layers, especially because different types of propagations (e.g., with phase delay) are used later in the manuscript.

The dynamics are propagated from one layer to the next, as the output from layer 1 is dynamic, meaning layer 2 receives a dynamic input. Moreover, the hidden activations in layer 2 also change according to the ordinary differential equations defined in equation 3 and 4, leading to a dynamic output. We have clarified this in 2.3 Network dynamics in the hidden layers, ll. 130

The input z_j to the ODEs in the second hidden layer was calculated from the dynamic outputs of the first layer, and the dynamic activations in the second layer were used to calculate z_j in the softmax activation

Equation (2). The result of this feedforward propagation from the first to the second hidden layer is explored in Figure 4 and 7.

Relatedly, it would be interesting to check the robustness and generalizability of the results across a range of similar architectures. In particular, in the architecture described in the manuscript, few possible images are allowed (3 letters x 4 quadrants), and the kernel is perfectly adapted to the size of the image (28x28), and a large stride is employed, effectively conveying independent input for each quadrant. This could potentially result in overfittng. I think it would be instructive to explore architectures with smaller, more common kernel sizes and stride (e.g., 3x3 or 5x5) to see how results generalize when the first layer learns more elementary features. In par3cular, I expect the second layer dynamics in Fig. 7 to exhibit less segregation, but this is just a guess.

We agree with the reviewer that an exploration of our ideas in a different architecture would be interesting. However, the scope of this manuscript was to present the idea of taking inspiration from the dynamics observed in electrophysiological recordings from the human and non-human primate visual system, to implement a multiplexed coding scheme in an ANN. We purposefully present a network with a reduced architecture that can solve a simple, tractable problem, such that we could thoroughly investigate the parameter space of the ODEs (Figure 3), as well as the dynamics in each layer (Figure 4) and the parallelization and segregation of the representations in the network (Figure 7). For this simple model, we present a total of 9 different experiments (plus the hyperparameter tuning) in 13 Figures. As such, we feel that an application in a deeper architecture would warrant a separate publication. The issue of overfitting has now been addressed by adding noise to the images (as per a request by reviewer 2) and using a newly generated test set for the simulations the network has not seen before.

We understand that we may have raised the expectation that the manuscript will explore the presented ideas in a deep CNN, due to the strong focus on convolutional networks in the abstract and introduction and the application of a convolutional kernel for weight-sharing.

Therefore, we clarified our objective and goals in the abstract, introduction, methods and hope that this addresses the reviewer's concern sufficiently.

ll. 1

The field of computer vision has long drawn inspiration from neuroscientific studies of the human and non-human primate visual system. The development of convolutional neural networks (CNNs), for example, was informed by the properties of simple and complex cells in early visual cortex. However, the computational relevance of oscillatory dynamics experimentally observed in the visual system are typically not considered in artificial neural networks (ANNs).

L. 20

The inclusion of convolution in artificial neural networks (ANNs) was originally inspired by the feature detection properties of cells in early visual cortex, and marked a significant milestone in computer vision [20].

l. 26

Despite the success of embracing the spatial tuning properties of visual neurons for computer vision, there are only few examples of ANNs that have drawn inspiration from the *temporal dynamics* of cortical activity [8, 28, 22].

l. 36

We deliberately present a tractable network with a reduced architecture to demonstrate the computational benefit of oscillatory dynamics in computer vision. Our aim is to pave the way for applications in deep CNNs that can benefit from both the spatial tuning properties and temporal dynamics of the visual system.

And the discussion l. 415

While the simple nature of the network limits its computational abilities, it allowed a tractable implementation and comprehensive exploration of the imposed dynamics, as demonstrated in Figure 3, 4, and 6. As such, the presented work sets the stage for applying the presented principles to CNNs with a deeper archiecture that can solve benchmark image classification problems such as (E)MNIST [6, 21], CIFAR-10 [19], and ImageNet [31].

Minor comments:

- If the task is classification, why using the mean squared error as training objective (fig. 2c)?

We agree with the reviewer that cross-entropy loss would have been a more appropriate choice for image classification. In the previous version of the manuscript the network was trained and tested on

the same, simple stimuli. For this simple problem, we found MSE loss to converge faster and lead to the somewhat binary activations we were aiming to achieve for our dynamics. We have now changed our training and test set based on a request by reviewer 2 and added low amplitude noise to each image. For this set, we found that cross-entropy loss performed well and are therefore using it for the new network (please see Figure 2b and c).

- There seems to be some inconsistency in Figure referencing, e.g., in page 3 both citations should be Figure 2, and similary in page 5, and 6 (last reference before the last paragraph). In page 8, paragraph 3.3.1, the reference should to Fig 5b.

Thank you, we have corrected these referencing mistakes.

- In Eq. (8), h_j seems to be a scalar, there is no need to define the Hadamard product **Yes, we agree with the reviewer and have updated equation (8)**

- The words refractory and relaxation dynamics are used interchangeably, I would s3ck to one choice to avoid confusions **We agree and now use the term "refraction" consistently throughout the manuscript.**

- I really enjoyed reading the discussion about the possible future directions on the neuroscientific and machine learning side. In this case, the dynamics was imposed after training; I wonder whether the authors could instead comment on possible training mechanisms that would give rise to such dynamics?

We have addressed this comment in the final paragraph of our discussion. While we agree that introducing dynamics into the training process may be interesting, we feel that the current implementation may be more realistic in the context of object recognition.

l. 436

Moreover, Liebe et al. [22] have demonstrated emerging oscillatory dynamics when training an RNN to memorise a sequence. A key difference to our work is that the inputs and outputs in these previous studies were dynamic, which may have resulted in emerging dynamics with minimal intervention by the researcher. However, it would still be interesting to test whether training a network to not only classify images but also to convert simultaneous stationary inputs into a sequence results in rhythmic activations in the hidden layers. It should be emphasised however, that we here provide an implementation of the idea that top-down control by

inhibitory alpha oscillations supports multiplexing. This oscillatory top-down control has been proposed to reach the sensory systems of the human and primate brain through thalamo-cortical connections [14] or as a backwards travelling wave initiated in frontal regions [10]. The logic of imposing the dynamics after the training was based on the notion that learning to recognise different objects, i.e. the main task of the visual ventral stream, is different to learning to represent items in a sequence. As such, we argue that the current implementation with imposed external top-down control to learned representations of visual objects is more biologically realistic for the presented problem.
